# Ca^2+^-stimulated adenylyl cyclases as therapeutic targets for psychiatric and neurodevelopmental disorders

**DOI:** 10.3389/fphar.2022.949384

**Published:** 2022-09-16

**Authors:** Jiao Chen, Qi Ding, Lulu An, Hongbing Wang

**Affiliations:** Department of Physiology, Michigan State University, East Lansing, MI, United States

**Keywords:** autism, Ca^2+^-stimulated adenylyl cyclase, bipolar disorder, schizophrenia, major depressive disorder, post-traumatic stress disorder, therapeutics

## Abstract

As the main secondary messengers, cyclic AMP (cAMP) and Ca^2+^ trigger intracellular signal transduction cascade and, in turn, regulate many aspects of cellular function in developing and mature neurons. The group I adenylyl cyclase (ADCY, also known as AC) isoforms, including ADCY1, 3, and 8 (also known as AC1, AC3, and AC8), are stimulated by Ca^2+^ and thus functionally positioned to integrate cAMP and Ca^2+^ signaling. Emerging lines of evidence have suggested the association of the Ca^2+^-stimulated ADCYs with bipolar disorder, schizophrenia, major depressive disorder, post-traumatic stress disorder, and autism. In this review, we discuss the molecular and cellular features as well as the physiological functions of ADCY1, 3, and 8. We further discuss the recent therapeutic development to target the Ca^2+^-stimulated ADCYs for potential treatments of psychiatric and neurodevelopmental disorders.

## Introduction

**FIGURE 1:**
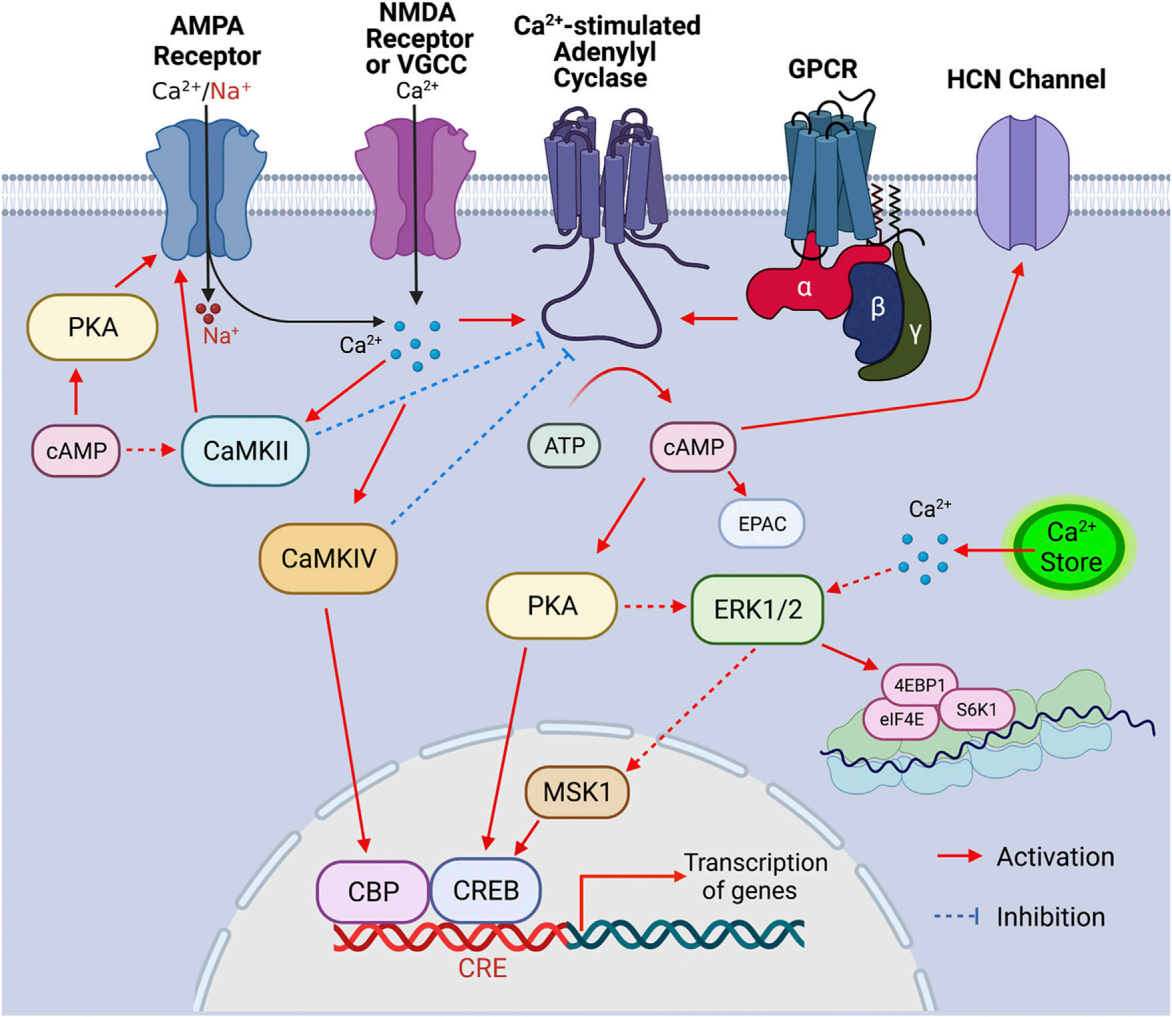
Signaling cascade triggered by the activation of Ca^2+^-stimulated ADCYs. Upon the activation of GPCR, Ca^2+^ influx through Ca^2+^ channels (such as VGCC, NMDAR, and Ca^2+^-permeable AMPAR) and/or Ca^2+^ release from internal store, the Ca^2+^-stimulated ADCYs are activated, leading to increased production of cAMP. The upregulation of cAMP activates PKA, which causes phosphorylation of CREB, ERK_1/2_ (indirectly), CaMKII (indirectly) and GluR1 (at S845), leading to activation of these molecules. Increase of intracellular Ca^2+^ leads to activation of ERK_1/2_ (indirectly), CaMKII, and CaMKIV, which, in turn, activate MSK1/CREB, GluR1 phosphorylation (at S831), and CBP (CREB binding protein), respectively. The activated CBP/CREB triggers CRE (cAMP responsive element)-mediated gene transcription. The ERK_1/2_-mediated activation of eIF4E, 4EBP1, and S6K1 may enhance ribosome activity and lead to increase of protein translation. The Ca^2+^-stimulated CaMKIV and CaMKII may lead to feedback inhibition of ADCY1 and ADCY3, respectively. Other main aspects of cAMP effect include the cAMP-mediated EPAC (exchange protein directly activated by cAMP) activation and cAMP-facilitated opening of the HCN (hyperpolarization-activated cyclic nucleotide-gated) channels.

Adenylyl cyclase (ADCY) activity accounts for the basal level as well as activity-dependent production of cAMP, which is a main second messenger and regulates a wide spectrum of intracellular signaling molecules (Figure 1). In the nervous system, extracellular stimuli lead to functional responses and activity-dependent plasticity through the activation of surface neurotransmitter receptors followed by activation/inhibition of intracellular signaling molecules. With its enzymatic activity directly regulated by the G protein coupled neurotransmitter receptors (GPCR) on cell membrane, ADCY contributes to the dynamic intracellular cAMP transient following extracellular stimulation. The outcome of the cAMP-mediated signal transduction has broad impact on cellular functions including gene transcription and translation (Figure 1), the precise regulation of which is essential for neurodevelopment and activity-dependent modification of neuronal function. Thus, it is envisioned that appropriate ADCY activity is critical for neurodevelopment and neuroplasticity. Further, in addition to GPCRs, many other intracellular signaling molecules also dynamically regulate ADCY activity (Figure 1), leading to fine tuning of cAMP signaling in a task-specific manner. With regards to their physiological function, specific isoforms of ADCY have been shown to regulate distinct aspects of neuroplasticity and behavior that are essential for survival and adaptation. Emerging lines of animal study and human genome data have also suggested association of ADCY with a variety of dysfunction in the central nervous system (CNS) and the peripheral systems.

To date, ten isoforms of mammalian ADCY have been identified. Nine ADCYs (i.e., ADCYs 1-9) are membrane-bound proteins and share similar structures with a short N-terminal cytoplasmic domain (the C1 domain) two transmembrane domains (i.e., TM1 and TM2) connected by the C1 cytoplasmic loop, and the C2 cytoplasmic domain at the C-terminus (Cooper, 2003) (Figure 1). Through the C1 and C2 domains, ADCYs 1-9 confer the catalytic activity and can be regulated by GPCRs and intracellular protein kinases and phosphatases (Cooper, 2003) (Figure 1). Based on structure and regulatory properties, five groups of ADCY are defined. In general, the group I ADCYs includes ADCY1, 3, and 8. They are stimulated by G_s_ and Ca^2+^ and inhibited by G_i_. The group II ADCYs include ADCY2, 4, and 7. They are stimulated by G_s_ and not responsive to Ca^2+^ and G_i_. The group III ADCYs include ADCY5 and 6. They are stimulated by G_s_ and inhibited by Ca^2+^ and G_i_. The group IV ADCY includes ADCY9, which is only stimulated by G_s_ and, unlike other ADCYs, not responsive to the general ADCY activator forskolin. The group V ADCY includes ADCY10, which is a structurally distinct soluble cytoplasmic protein whose activity is regulated by Ca^2+^ and bicarbonate but not GPCRs (Buck et al., 1999; Litvin et al., 2003; Kleinboelting et al., 2014). ADCY10 is mainly expressed in the liver and testes but not in the central nervous system. Among these ADCYs, the group I Ca^2+^-stimulated ADCYs are functionally positioned to couple the two main small molecule messengers Ca^2+^ and cAMP in neurons (Figure 1). Human genetic studies and animal models suggest emerging roles of the Ca^2+^-stimulated ADCYs in regulating distinct aspects of pathology in psychiatric disorders. In this review, we focus on the Ca^2+^-stimulated ADCYs and discuss their potential as therapeutic targets to treat neurodevelopment and psychiatric disorders.

## Regulation and molecular targets of the Ca^2+^-stimulated adenylyl cyclases

### Regulation by Ca^2+^ and G proteins

The existence of the calmodulin (CaM)-binding domain in the C1 or C2 region of some ADCYs suggests that certain isoforms can be regulated by Ca^2+^ (Wang and Storm, 2003). Affinity purification with the CaM-sepharose chromatography aided the cloning of *ADCY1* followed by discovering other CaM-binding ADCYs (Krupinski et al., 1989). Among all the Ca^2+^-sensitive ADCYs, the group I isoforms consists of the Ca^2+^-stimulated ADCY1, ADCY3 and ADCY8 (Cooper, 2003). As cAMP and Ca^2+^ are the main second messengers in neurons, it is postulated and the Ca^2+^-stimulated ADCYs are at the converging hub to integrate the cAMP and Ca^2+^ signaling (Figure 1).

ADCY1 activity is stimulated by Ca^2+^ (Tang et al., 1991) with an EC50 of ∼100 nM (Fagan et al., 1996), which is slightly lower than the free Ca^2+^ level in resting neurons (Berridge et al., 2003). This indicates that ADCY1 is constitutively stimulated by the basal Ca^2+^ in resting neurons and further activated by transient increase of Ca^2+^ in stimulated neurons. Distinct amino acid mutations in the CaM-binding domain either reduce or abolish the Ca^2+^ stimulation of ADCY1 (Wu et al., 1993). Although GTPγS can directly increase ADCY1 activity (Tang et al., 1991), G_αs_ only stimulates ADCY1 in the presence of Ca^2+^ (Wayman et al., 1994); G_αi_ (Nielsen et al., 1996) and G_βγ_ (Tang et al., 1991) cause inhibition of ADCY1. Intriguingly, CaMKIV (CaM-dependent kinase IV) can directly phosphorylate ADCY1, leading to activity inhibition (Wayman et al., 1996). It is conceivable that, depending on subcellular micro-environment, Ca^2+^/CaM may exert dichotomy effects on ADCY1. While Ca^2+^ may predominantly stimulate ADCY1 in sub-cellular regions that are devoid of CaMKIV, a compromised or even inhibitory effect may be observed in the CaMKIV-enriched regions. CaMKIV may also function as a molecular break to prevent over-stimulation of ADCY1 by sustained Ca^2+^ elevation (Figure 1).

ADCY3 shares the most structural similarity with ADCY1 and ADCY8. However, *in vitro* assays with membrane preparation reveal that Ca^2+^ alone is not sufficient to stimulate ADCY3 activity (Choi et al., 1992; Wayman et al., 1995). When paired with the general ADCY activator forskolin or G protein activator GppNHp, high level of Ca^2+^ stimulates ADCY3 with an EC50 of ∼5,000 nM (Choi et al., 1992). Considering that the intracellular Ca^2+^ varies in the 1–10,000 nM range (Smith and Augustine, 1988), Ca^2+^ stimulation of ADCY3 may occur in specific subcellular domains with high Ca^2+^ transients in stimulated neurons. Notably, Wayman et al. found that, in the presence of GppNHp or glucagon, Ca^2+^ at a much lower concentration (e.g., 200 nM) can cause ∼2-fold increase of ADCY3 activity *in vitro* (Wayman et al., 1995). Counterintuitively, cAMP accumulation assay with live cells reveal that increase of intracellular Ca^2+^ (at ∼100–300 nM) significantly inhibits hormone- and G_αs_-stimulated ADCY3 *in vivo* (Wayman et al., 1995). It is further identified that CaMKII (CaM-dependent protein kinase II) phosphorylates and inhibits ADCY3, suggesting an indirect inhibition effect of Ca^2+^ (Wei et al., 1996) (Figure 1). Although the physiological relevance of the Ca^2+^-stimulated regulation of ADCY3 remains to be elucidated, the Ca^2+^/CaMKII-mediated inhibition is likely to ensure a transient rather than persistent increase of cAMP increase in olfactory cilia, leading to the temporal attenuation of sensation following odor detection (Wei et al., 1998).

ADCY8 possesses a CaM-binding domain and is directly stimulated by Ca^2+^ with an EC50 of ∼500–800 nM (Fagan et al., 1996; Nielsen et al., 1996). Considering that ADCY8 is moderately sensitive to Ca^2+^, it is speculated that Ca^2+^ released from the internal store may not be sufficient to stimulate ADCY8. However, Martin et al. (2009) suggest that the capacitative Ca^2+^ entry (CCE), which is triggered by Ca^2+^ depletion from the internal store, may lead to more robust Ca^2+^ increase and stimulate ADCY8. In the non-neuronal HEK293 cells, when ADCY8 and the CCE functional molecules STIM1 and Orai1 are over-expressed, they colocalize in lipid raft domains of the cell membrane. Functionally, while Ca^2+^ store depletion alone fails to activate ADCY8, over-expression of STIM1 and Orai1 along with Ca^2+^ store depletion leads to ADCY8 activation (Martin et al., 2009). Given that there is high expression level of STIM and Orai isoforms in the central nervous system (e.g., STIM2 and Orai2 in hippocampus), it is likely that CCE activates ADCY8 as well as ADCY1 and ADCY3 in neurons (Zhang and Hu, 2020). *In vitro* assays with membrane preparations from ADCY8-expressing HEK293 cells detect that the Ca^2+^-stimulated ADCY activity can be further increased in the presence of activated G_αs_ (i.e., GTPγS bound G_αs_) (Cali et al., 1994). However, the *in vivo* cAMP accumulation assay fails to detect any effect in HEK293 cells following pharmacological activation of the Gs-coupled adrenergic receptors (Cali et al., 1994; Nielsen et al., 1996). Further, the *in vivo* cAMP accumulation assay with the ADCY8-expressing HEK293 cells fails to detect the inhibition effects following the activation of the Gi-coupled somatostatin and dopaminergic receptors (Nielsen et al., 1996). Thus, these lines of evidence suggest that ADCY8 is exclusively regulated by Ca^2+^ but not by G proteins *in vivo*. However, overexpression of a constitutively active G_α/olf_ causes robust activation of both ADCY1 and ADCY8 (Regnauld et al., 2002). Regarding whether and how GPCRs can regulate ADCY8 activity, examination with neurons (rather than the heterologous HEK293 cells) and activation of full spectrum of GPCRs (in addition to adrenergic, dopaminergic and somatostatin receptors) is required.

### Molecular targets

Through the Ca^2+^-stimulated ADCYs, cAMP and Ca^2+^ signaling may converge and tune specific signaling networks and, in turn, regulate cellular functions relevant to neurotransmitter receptor activity, gene transcription, and translation (Figure 1). With regards to the function of Ca^2+^-stimulated ADCYs in neuroplasticity, neurodevelopment and psychiatric disorders, we focus on the Ca^2+^/cAMP-PKA (cAMP-dependent protein kinase)/ERK_1/2_ (extracellular signal-regulated kinases 1/2)-MSK1 (mitogen- and stress-activated protein kinase-1)-CREB (cAMP responsive element binding protein), the Ca^2+^/cAMP-ERK_1/2_-eIF4E (eukaryotic translation initiation factor 4E)/4EBP1 (Eukaryotic translation initiation factor 4E-binding protein 1)/S6K1 (ribosome protein S6 kinase 1), and the Ca^2+^/cAMP-PKA/CaMKII-GluR1 (glutamate ionotropic AMPA type subunit 1) cascades (Figure 1).

The definitive function of ADCY1 and ADCY8 in regulating the Ca^2+^/cAMP-mediated signaling is mainly examined with brain samples collected from mutant mice. The Ca^2+^-stimulated ADCY activity is reduced by ∼50% and ∼30% in the hippocampus of *Adcy1* knockout (KO) and *Adcy8* KO mice, respectively (Wong et al., 1999). The cAMP level is reduced by ∼25% in the *Adcy1* KO hippocampus (Sethna et al., 2017). Although lack of both ADCY1 and ADCY8 surprisingly increases the basal ERK1/2 activity, the contextual fear learning- and cocaine-triggered upregulation of the ERK_1/2_-MSK1-CREB signaling is abolished in the *Adcy1*/*Adcy8* double knockout (DKO) mice (Sindreu et al., 2007; DiRocco et al., 2009). The *Adcy1*/*Adcy8* DKO mice also do not display diurnal oscillation of ERK_1/2_ phosphorylation in the hippocampus (Eckel-Mahan et al., 2008), suggesting lack of molecular circadian rhythm. In primary cortical neurons, ADCY1 deficiency impairs the glutamate-induced upregulation of CREB activity (Wang et al., 2007). Consistent with the function of ERK_1/2_-CREB in regulating the Ca^2+^/CRE (cAMP-responsive element)-mediated transcription of *bdnf* (brain-derived neurotrophic factor) (Zheng et al., 2011), mice lacking ADCY1 and ADCY8 fail to show learning- and exercise-induced up-regulation of *bdnf* mRNA (Zheng et al., 2012; Zheng et al., 2016). Conversely, overexpression of ADCY1 results in increase of Ca^2+^-stimulated ADCY activity, cAMP level, PKA activity, and basal as well as learning-induced upregulation of ERK_1/2_-CREB activity in the hippocampus (Wang et al., 2004). Overexpression of ADCY1 also blocks the stress-induced downregulation of *bdnf* transcription in hippocampus and prefrontal cortex (Yang et al., 2020).

With regards to gene expression, the cAMP-regulated ERK_1/2_ may impinge on ribosome activity and, in turn, regulate protein synthesis. *In vitro* and *in vivo* studies demonstrate that inhibition of ERK_1/2_ leads to impairments of activity-dependent upregulation of eIF4E, 4EBP1, and S6K1 phosphorylation (Kelleher et al., 2004; Zhou et al., 2010). Inhibition of ERK_1/2_ also suppresses various forms of protein synthesis-dependent synaptic plasticity (Gallagher et al., 2004; Kelleher et al., 2004). Whether and how the Ca^2+^-stimulated ADCYs regulate basal and activity-dependent changes of ribosome activity remain to be determined.

The cAMP signaling may also regulate neuronal function on cell surface. Two main phosphorylation sites (i.e., Serine 845 and 831) of GluR1 are targets of PKA and CaMKII/PKC (protein kinase C), respectively (Figure 1), and functionally involved to regulate receptor trafficking and channel conductance (Malinow, 2003). The phosphorylation of GluR1 at S845 and S831 is dynamically altered after the induction of long-term potentiation (LTP), long-term depression (LTD.), and synaptic depotentiation (Lee et al., 2000; Huang et al., 2001). Although S831 phosphorylation can be suppressed by PKC and CaMKII inhibitors, it may also be regulated indirectly through the cAMP-enhanced CaMKII activation (Makhinson et al., 1999). In mice with a natural loss-of-function mutation in *Adcy1*, phosphorylation of GluR1 (pGluR1) at S845, AMPAR-mediated EPSCs, and surface GluR1 are decreased at the thalamocortical synapses (Lu et al., 2003).

## Expression pattern and cellular function of the Ca^2+^-stimulated adenylyl cyclases

### Adenylyl cyclase1

**FIGURE 2:**
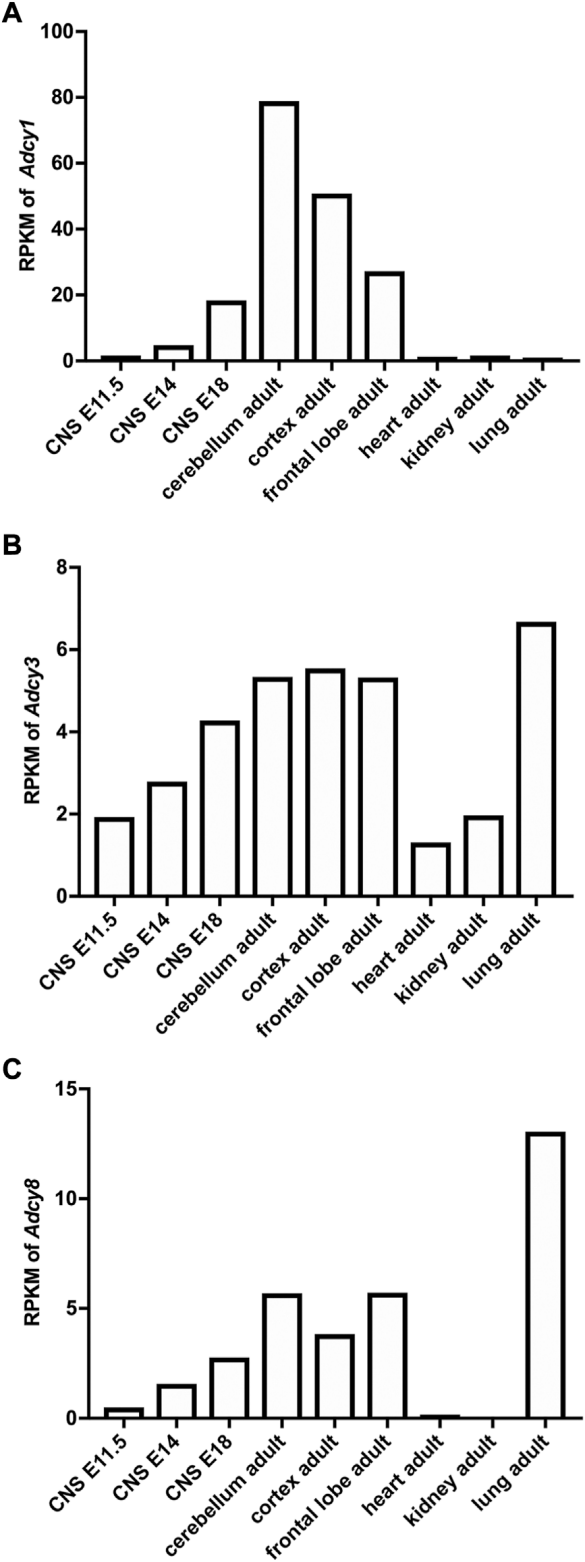
Developmental and tissue-specific expression of Ca^2+^-stimulated *Adcy* mRNA transcripts in mouse. The RNAseq study by the Mouse ENCODE Consortium (Yue et al., 2014) revealed the expression of *Adcy1* **(A)**, *Adcy3* **(B)**, and *Adcy8* **(C)** mRNA in different tissues and in CNS at different developmental stages. RPKM: Reads Per kilobase of transcript per Million mapped reads. CNS: central nervous system. E11.5, E14, and E18: embryonic day 11.5, 14, and 18.

**FIGURE 3:**
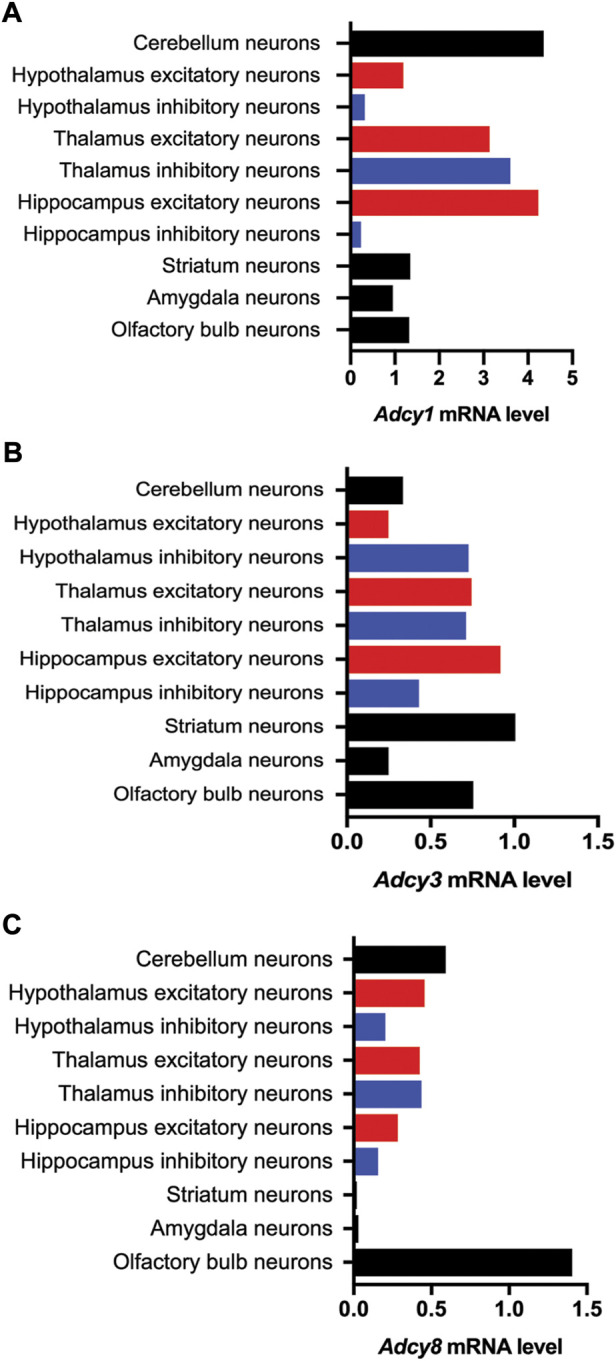
Cell type-specific expression of Ca^2+^-stimulated *Adcy* mRNA transcripts in various regions of the mouse brain. The information of relative mRNA levels of *Adcy1* **(A)**, *Adcy3* **(B)**, and *Adcy8* **(C)** in excitatory and inhibitory neurons of various brain regions was collected from https://mousebrain.org.

The mRNA transcript of *Adcy1,* detected by Northern blot and *in situ* hybridization, is predominantly expressed in the nervous system tissues including brain, adrenal gland, and retina (Figure 2A) (Xia et al., 1993; Yue et al., 2014). Within the central nervous system (CNS), *Adcy1* mRNA expression is developmentally regulated (Figure 2A) and detected in distinct regions at various levels (Figures 2A, 3A) (Yue et al., 2014) (https://brainrnaseq.org). Notably, *Adcy1* mRNA level is overwhelmingly higher in excitatory neurons than inhibitory neurons in the hippocampus but not in other regions (Figure 3A). Although RNA-sequencing has detected *Adcy1* mRNA in neurons and glial cells (https://brainrnaseq.org), Western blot with a validated antibody (Sethna et al., 2017) detects ADCY1 protein expression only in neuron-enriched but not glial cell-enriched primary cultures (Ding et al., 2020).

The subcellular distribution of endogenous ADCY1, due to the lack of antibody for histochemistry, is largely unknown. With expression of the epitope-tagged recombinant protein, it is found that the HA (hemagglutinin)-ADCY1 is expressed in both dendrite and axon. The punctate and discrete HA-ADCY1 colocalizes with synaptophysin and synapsin I in cerebellar neurons, indicating enrichment in the synaptic compartment (Wang et al., 2002). Western blot analysis with synaptosome fractions detects that the endogenous ADCY1 in hippocampus is enriched in postsynaptic density and extrasynaptic fractions (Conti et al., 2007).

Consistent with the high expression level of *Adcy1* mRNA in cortex, hippocampus, and cerebellum, studies with the *Adcy1* KO mice have found that activity-dependent potentiation of synaptic efficacy in these brain regions requires ADCY1 (Storm et al., 1998; Liauw et al., 2005; Zheng et al., 2016). It is also evident that ADCY1 supports long-term potentiation (LTP) at both presynaptic (Villacres et al., 1998; Miao et al., 2019) and postsynaptic sites (Liauw et al., 2005; Zheng et al., 2016). It is demonstrated that ADCY1 in presynaptic neurons is required for sensory input and neurodevelopment of postsynaptic neurons. Brain region-specific knockout of *Adcy1* in thalamus causes disruptive barrel patterning in layer 4 of the somatosensory cortex (Suzuki et al., 2015). Further, ADCY1 deficiency in the presynaptic RGC (retinal ganglion cell) causes map disruption of the postsynaptic tissues SC (superior colliculus) and LGN (lateral geniculate nucleus) (Dhande et al., 2012).

### Adenylyl cyclase3

ADCY3 was initially identified as the major ADCY in olfactory epithelium (Bakalyar and Reed, 1990). Following the molecular cloning of *Adcy3* gene, Northern blot and semiquantitative RT-PCR detected broad expression of the *Adcy3* mRNA in both CNS and peripheral tissues (Xia et al., 1992; Yang et al., 1999; Yue et al., 2014) (Figure 2B). The expression level is high in brain, placenta, lung, and skeletal muscle. Intermediate expression is detected in heart, kidney, and pancreas (Yang et al., 1999; Yue et al., 2014). Within the CNS, *Adcy3* mRNA and ADCY3 protein are found in many brain regions, including olfactory bulb, cortex, hippocampus, amygdala, nucleus accumbens, thalamus, hypothalamus and cerebellum (Bishop et al., 2007) (Figures 2B, 3B). It is expressed in both excitatory and inhibitory neurons (Figure 3B) as well as in glia cells (Bishop et al., 2007). Interestingly, *Adcy3* mRNA level is higher in inhibitory neurons than excitatory neurons in hypothalamus (Figure 3B).

Within the olfactory epithelium, ADCY3 is predominantly localized in olfactory cilia, which is the main organelle of sensory neuron to conduct the sensation of smell. Upon activation of the G_olf_-coupled odorant receptors, cAMP is generated by the G_olf_-activated ADCY3. Binding of cAMP to the cyclic nucleotide-gated (CNG) channels causes the influx of Na^+^ and Ca^2+^, leading to sensory neuron activation and the subsequent olfactory detection process. Deficiency of ADCY3, which is the only ADCY in olfactory cilia, causes anosmia (i.e., loss of smell) (Wong et al., 2000).

In brain neurons, ADCY3 is localized in the primary cilium (Bishop et al., 2007), which is a solitary microtubule-based 2–12 μm projection from the cell surface. In contrast to typical synaptic structures, primary cilia are devoid of ionotropic neurotransmitter receptors and thought to mediate neuronal signaling via metabotropic GPCRs (Green and Mykytyn, 2014). The co-existence of ADCY3 and certain GPCRs (e.g., melanocortin 4 receptor, somatostatin receptor 3, and type 6 serotonin receptor) suggests activity-dependent cAMP signaling in primary cilia (Wang et al., 2011b; Guadiana et al., 2013; Siljee et al., 2018). Although how cilia signaling affects neuronal function is largely unknown, disruption of the cilia-enriched proteins causes alteration of neuron development and synaptic function. Notably, ADCY3 deficiency leads to reduced dendritic outgrowth and arborization, hippocampus atrophy, reduced neural transmission and impaired LTP at the schaffer collateral-CA1 synapses (Chen et al., 2016).

### Adenylyl cyclase8

*Adcy8* is expressed in both the CNS and the peripheral non-neuronal tissues (Muglia et al., 1999; Yue et al., 2014) (Figure 2C). Within the CNS, *Adcy8* mRNA shows the highest levels in olfactory bulb, thalamus, hypothalamus and brain stem (Muglia et al., 1999; Schaefer et al., 2000) (Figure 3C). In these brain regions, ADCY8 but not ADCY1 accounts for most of the Ca^2+^-stimulated ADCY activity. In olfactory bulb and hypothalamus of the *Adcy8* KO mice, there is no significant Ca^2+^-stimulated upregulation of ADCY activity (Schaefer et al., 2000). Moderately high level of *Adcy8* mRNA is detected in hippocampus, in which ADCY8 deficiency causes ∼25% reduction of Ca^2+^-stimulated ADCY activity (Schaefer et al., 2000). In contrast to *Adcy1* mRNA, robust *Adcy8* mRNA is detected in both excitatory and inhibitory neurons in hippocampus (Figure 3C). This suggests that the Ca^2+^-stimulated cAMP signaling may be co-regulated by ADCY1 and ADCY8 in excitatory neurons, and predominantly regulated by ADCY8 in inhibitory neurons in the hippocampus.

The subcellular distribution of ADCY8 has been examined with neurons expressing the recombinant HA-ADCY8. The HA-ADCY8 displays punctate staining in both dendrites and axons of cortical and hippocampal neurons and colocalizes with both presynaptic (i.e., synaptophysin and synapsin I) and postsynaptic marker (i.e., PSD95) proteins (Wang et al., 2003). The endogenous hippocampal ADCY8 is preferentially enriched in the presynaptic active zone and also detected in extrasynaptic fractions (Conti et al., 2007). The presynaptic cellular function of ADCY8 is implicated by that the mossy fiber-CA3 LTP, which mainly relies on presynaptic Ca^2+^/cAMP signaling, is defective in the *Adcy8* KO mice (Wang et al., 2003). The postsynaptic function of ADCY8 is implicated by that the schaffer collateral-CA1 LTD (long-term depression) is defective in the *Adcy8* KO mice (Schaefer et al., 2000).

## Association of the Ca^2+^-stimulated adenylyl cyclases with psychiatric and neurodevelopment disorders

Alteration of Ca^2+^/cAMP-mediated signaling has been detected as molecular outcomes that are associated with various aspects of psychiatric and neurodevelopment disorders. Within the Ca^2+^/cAMP signaling network (Figure 1), abnormal function of GPCR (Grace, 2016), ion channel (Lee et al., 2016; Zanos and Gould, 2018; Nakazawa and Sapkota, 2020), and protein kinase (Wang et al., 2012; Robison, 2014; Gross et al., 2019) is associated with distinct malfunction and maladaptation of the brain. Here, we discuss the emerging roles of Ca^2+^-stimulated ADCY as risk and causal factors in regulating the cellular pathology and behavioral symptoms associated with psychiatric and neurodevelopment disorders.

### Adenylyl cyclase1

Hyper-expression of ADCY1 has been identified in a mouse model of Fragile X syndrome (FXS) (Sethna et al., 2017), which is predominantly caused by mutations in the *FMR1* (Fragile X messenger ribonucleoprotein 1) gene and deficient expression of its gene product FMRP (FMR1 protein). FXS is a neurodevelopment disorder and leading cause of intellectual disability and autism (Sethna et al., 2014). Among various functions of FMRP (Richter and Zhao, 2021), the RNA binding activity has been demonstrated to be directly related to the main symptoms in FXS. High-throughput screenings have revealed that FMRP binds 800–6,000 distinct mRNA targets and may suppress their translation (Brown et al., 2001; Darnell et al., 2011; Ascano et al., 2012). Along with general elevation of mRNA translation, abnormally increased expression of specific FMRP targets is linked to exaggerated signaling in FXS neurons (Wang et al., 2012; Gross et al., 2015). Mining and analysis of the multiple high-throughput screening data identified *Adcy1* mRNA as a top-ranked FMRP target. Consistently, ADCY1 protein expression level is abnormally higher in the brain of *Fmr1* knockout (KO) mice (Sethna et al., 2017). The enhanced ADCY1 expression is associated with the exaggerated ERK_1/2_/PI3K (phosphoinositide 3-kinases)-S6K1 signaling in FXS (Wang et al., 2012; Gross et al., 2015). The causal function of the elevated ADCY1 expression is supported by that genetic reduction of ADCY1 in the *Fmr1* KO mice rescues the key aspects of pathology, including the exaggerated overall protein synthesis and ERK_1/2_/PI3K-S6K1 activity, higher dendritic spine density, audiogenic seizure, repetitive behavior and social deficits (Sethna et al., 2017). Interestingly, enhanced ADCY1 expression also results in certain behavioral abnormalities associated with FXS and autism. Forebrain overexpression of ADCY1 in transgenic mice causes hyper locomotion and social deficits (Chen et al., 2015).

Alteration of *Adcy1* gene, as suggested by the human genome-wide association study (GWAS), is a potential genetic risk factor for schizophrenia (Sundararajan et al., 2018). A recent study analyzed a combined list of schizophrenia-risk genes that are collected from published GWAS data, meta-analysis data, and the OMIM and GeneCards databses (Butler et al., 2016a; Butler et al., 2016b; Wu et al., 2017). By using the GeneAnalytics program, Sundararajan et al. (2018) suggest that the schizophrenia genes have significant impact on Ca^2+^ signaling pathway, CREB pathway, and monoamine GPCRs. Integration analysis of the schizophrenia gene and the phenotype database (http://www.informatics.jax.org/; http://human-phenotype-ontology.github.io/) identifies an association of schizophrenia genes with reduced LTP, abnormal spatial learning, and neurodevelopment (Sundararajan et al., 2018). Based on biochemical data and functional studies with mutant mice, it is evident that ADCY1 directly regulates the CREB pathway and integrates Ca^2+^ and GPCR signaling. Although how ADCY1 regulates the hallmark schizophrenia symptoms remains unclear, ADCY1 deficiency leads to impaired LTP (Villacres et al., 1998; Zheng et al., 2016) and spatial memory (Wu et al., 1995) and maldevelopment of the sensory cortex (Suzuki et al., 2015). It is interesting to note that ADCY1 shows high level in cerebellum, cerebral cortex, and thalamus, which are where the schizophrenia risk genes predominantly express (Sundararajan et al., 2018). As defective cortico-cerebellar-thalamic-cortical circuit is suggested as an emerging etiological factor for schizophrenia (Andreasen and Pierson, 2008; Dorph-Petersen and Lewis, 2017), the region-specific function of ADCY1 needs to be studied.

Alterations of *Adcy1* gene may affect the therapeutic efficacy of lithium in bipolar disorder (International Consortium on Lithium Genetics et al., 2018). Although bipolar disorder and schizophrenia shares significant number of genetic risk factors, patient responses to pharmacological interventions are dramatically different. While the mood stabilizer lithium is used as the first line medication in bipolar disorder, it is not effective for schizophrenia patients. Further, a significant population of bipolar disorder patients also does not show therapeutic outcome following lithium treatment. A cross-trait meta-analysis of the GWAS of schizophrenia and Consortium on Lithium Genetics has found an interesting reverse association of polygenic schizophrenia load and lithium response in bipolar disorder (International Consortium on Lithium Genetics et al., 2018). Bipolar disorder patients with low polygenic schizophrenia load show better therapeutic response to lithium. Regarding whether and how ADCY1 activity impinges on pharmacological and molecular outcome of lithium treatment, validations with *in vitro* cellular assays and *in vivo* behavioral examinations may be needed. With the available ADCY1 inhibitors and mouse models (e.g., the *Adcy1* KO and overexpression mice), it is feasible to detect whether the lithium effects are attenuated or potentiated.

### Adenylyl cyclase3

Although there are debates on the significance of genetic risk factors in human major depressive disorder, a GWAS study with 2,431 major depressive disorder and 3,673 control samples revealed a suggestive association of *Adcy3* polymorphism with depression (Wray et al., 2012). Notably, lower PKA expression and ADCY activity (Perez et al., 2001; Hines et al., 2005) in platelets, which express only *Adcy3* but not other *Adcy* isoforms (Katsel et al., 2003), are detected in major depressive disorder subjects and attenuated by the use of various drugs including antidepressants, analgesics, and addictive drugs (Hines et al., 2005). Consistently, *Adcy3* mRNA level is reduced in the blood samples of major depressive disorder (Redei et al., 2014). Brain transcriptome analysis of human postmortem samples has also found altered level of *Adcy3* transcript in autism spectrum disorder (Guan et al., 2019).

The functional relevance of *Adcy3* mutations to major depressive disorder and autism spectrum disorder has been examined with the KO and knock-in mice. The conventional whole body *Adcy3* KO mice display a wide spectrum of symptoms including increased REM (rapid eye movement) sleep, hypo-locomotion, neophobia, higher immobility in the tail suspension and forced swimming test, impaired nesting behavior and impaired sociability (Chen et al., 2016). The forebrain-specific pyramidal neuron conditional *Adcy3* KO mice recapitulates many aspects of the major depressive disorder- and autism spectrum disorder-associated symptoms, but intriguingly display normal social interaction (Chen et al., 2016). The conventional and the conditional forebrain-specific *Adcy3* KO mice show defective spatial memory and object recognition memory (Wang et al., 2011b; Chen et al., 2016). Whole body *Adcy3* deficiency also causes impairments in passive avoidance memory and fear memory extinction (Wang et al., 2011b). Intriguingly, the region-specific knock-down of *Adcy3* in the main olfactory epithelium not only leads to anosmia but also causes depression-like behavior (Liu et al., 2020a) and cognitive defects (Liu et al., 2020b). The results suggest a role of olfactory cAMP signaling in the association of olfaction deficiency and depression (Kohli et al., 2016); further understanding of how ADCY3 mediates the functional cross-talk among brain circuitry networks of olfaction, emotion and cognition is critical.

Further, *Adcy3* loss-of-function variants are identified as a risk factor of obesity (Grarup et al., 2018), which often co-occurs with depression (Carey et al., 2014; Mannan et al., 2016). Whole body KO or knockdown of *Adcy3* in ventromedial hypothalamus causes obesity (Wang et al., 2009; Yang et al., 2022). In contrast, mice overexpressing the human *ADCY3* gene (Yang et al., 2022) and mice harboring a point mutation, which causes elevated enzymatic activity of ADCY3, (Pitman et al., 2014) are resistant to high fat diet-induced obesity.

### Adenylyl cyclase8

Genetic variants of *Adcy8* gene, as suggested by the human genetics studies, may be associated with bipolar disorder, schizophrenia, autism spectrum disorder, obsessive-compulsive disorder, and posttraumatic stress disorder. A linkage study with microsatellite markers reported an association of bipolar disorder with the genetic loci on chromosome 8q24, which covers three candidate risk genes including *Adcy8* (Avramopoulos et al., 2004). Two follow-up studies using more SNP (single nucleotide polymorphism) markers revealed a finer mapping of the bipolar disorder-associated region on 8q24 and also suggest *Adcy8* as a potential risk gene (Zandi et al., 2008; Zhang et al., 2010). Brain transcriptome analysis of human postmortem samples has found altered level of *Adcy8* transcript in bipolar disorder and schizophrenia (Guan et al., 2019).

Clinical data have found that significant population of autism spectrum disorder and obsessive-compulsive disorder patients show overlapping pathological features (Gross-Isseroff et al., 2001; van Steensel et al., 2011). GWAS data also reveal genetic variants commonly associated with autism spectrum disorder and obsessive-compulsive disorder (Liu et al., 2019). Further, an integrative analysis of brain transcriptome in autism spectrum disorder and genomic variants in obsessive-compulsive disorder identifies *Adcy8* as a common risk factor for autism spectrum disorder and obsessive-compulsive disorder (Liu et al., 2019).

Regarding functional relevance of ADCY8 alteration to symptoms associated with psychiatric and neurodevelopmental disorders, supporting data are mostly collected from mice with *Adcy8* mutations. In mice with a QTL (quantitative trait loci) on a chromosome region that is homologous to human 8q24, increased *Adcy8* mRNA level is detected in ventral hypothalamus and piriform cortex and associated with avoidance behavior (i.e., preference of the sheltered feeding platform over the exposed feeding platform) (de Mooij-van Malsen et al., 2009). In contrast, the *Adcy8* KO mice display risk-taking traits, as indicated by more occupancy in the center area of the open field and open arm of the elevated plus maze (Schaefer et al., 2000). The *Adcy8* KO mice are also hyperactive in their home cage and during the forced swimming test (Razzoli et al., 2010).

The high expression level of *Adcy8* in hypothalamus suggests potential functions in regulating stress responses through the HPA (hypothalamic-pituitary-adrenal) axis. A GWAS study implicates a suggestive association of *Adcy8* with posttraumatic stress disorder (Wolf et al., 2014). *Adcy8* deficiency in the KO mice causes adrenal hypertrophy but normal basal plasma corticosterone level (Schaefer et al., 2000; Razzoli et al., 2010). Intriguingly, following chronic stress, the *Adcy8* KO mice display higher elevation of corticosterone (than wild type mice) along with more risk-taking rather than anxiogenic behavior (Schaefer et al., 2000; Razzoli et al., 2010).

## Development of therapeutic compounds

To achieve the therapeutic potential of targeting the Ca^2+^-stimulated ADCYs, it is necessary to develop pharmacological reagents. To date, several small molecule inhibitors against ADCY1 have been developed and tested in certain animal models. Drugs showing specific regulatory activity for ADCY3 and ADCY8 have not been reported yet.

By using a structure-based rational design compound library, Wang et al. (2011a) characterized and identified NB001 as a preferential inhibitor against ADCY1 over other ADCY isoforms. The peripherally administered NB001 can cross the blood-brain-barrier and have a half-life of about 2 h in the brain (Sethna et al., 2017). Although NB001 has a modest IC50 of 10 μM, a reasonably low dose at 1–3 mg/kg shows analgesic effect in preclinical models of neuropathic and inflammatory pain (Wang et al., 2011a). In a mouse model of FXS, NB001 at 1 mg/kg attenuates the abnormally elevated ERK_1/2_/Akt-S6K1 signaling and rescues repetitive behaviors and social deficits (Sethna et al., 2017).

Although NB001 is currently tested for safety in human clinical trials (Wang et al., 2022), it is not yet approved by FDA. The promising efficacy of NB001 with the FXS mouse model has motivated repurposing of the existing FDA-approved drugs. Ding et al. (2020) examined the effects of carbamazepine, which is an FDA-approved anticonvulsant and also shows pharmacological inhibition action against ADCY1 (Mann et al., 2009). It is demonstrated that carbamazepine attenuates the elevated ERK_1/2_/Akt activity and protein synthesis in the *Fmr1* KO neurons. Peripheral administration of carbamazepine corrects hyperlocomotion and social deficits and improves learning and memory in the *Fmr1* KO mice (Ding et al., 2020).

Another ADCY1 inhibitor has been identified by screening a natural product derivatives library. ST034307 shows an IC50 of 2.3 μM against ADCY1 and no detectable inhibition against other ADCYs (Brust et al., 2017). Interestingly, ST034307 at higher concentration (e.g., 30 μM) causes potentiation effect on ADCY2, 3, 5, and 6 (Brust et al., 2017). Consistent with the potential role of ADCY1 in pain (Li et al., 2020), intrathecal injection of ST034307 relieves pain in a mouse model of inflammatory pain (Brust et al., 2017).

As the backbone structures of the adenosine-based NB001 and chromone-based ST034307 predict limitation and drawback of these compounds as practical therapeutic reagents, recent effort aims to identify ADCY1 inhibitors with different structures. The oxadiazole-based AC10065 at micromolar concentrations can suppress both ADCY1 and ADCY8 (Kaur et al., 2019). New drug screening followed by structure optimization has revealed several pyrimidinone-based compounds that show selectivity of ADCY1 over other ADCYs with an IC50 at the sub-micromolar level (Scott et al., 2022). As these newly identified ADCY1 inhibitors show moderate therapeutic efficacy in an inflammatory pain model (Scott et al., 2022), further optimization may be needed.

## Conclusion and future directions

In summary, previous studies have demonstrated the function of Ca^2+^-stimulated ADCYs in regulating various aspects of neuronal property and behavior. It is evident that a distinct ADCY isoform, rather than general and overall cAMP level, may specifically control an isoform-specific cellular and physiological function. Elucidation of Ca^2+^-stimulated ADCY function in distinct brain regions and distinct cell types may help to develop precise intervention of the disorder-specific pathology. Development of high affinity isoform-specific drugs with favorable pharmacokinetics and toxicity profile will lead to practical intervention to promote mental health and alleviate symptoms associated with certain psychiatric and neurodevelopment disorders. Further, interpretation with the current animal models should consider non-specific effects of global gene deficiency, complication of different genetic backgrounds and validity of the behavior outcome. Precision preclinical models with more direct face and construct validity need to be developed.
